# Correlation of 3D Echocardiography With Cardiac MRI in Patients With Acute Left Ventricular Dysfunction in the Age Group of One Month to 18 Years

**DOI:** 10.7759/cureus.98246

**Published:** 2025-12-01

**Authors:** Varsha Shrivastava, Deen Dayal Nagar, Yamini Batham, Amit Kumar

**Affiliations:** 1 Pediatric Cardiology, Fortis Escorts Heart Institute, New Delhi, IND

**Keywords:** echocardiography, myocarditis, stroke volume, three-dimensional, ventricular dysfunction, ventricular function

## Abstract

Objectives

The objective of this study is to evaluate the correlation and agreement between three-dimensional echocardiography (3D-ECHO) and cardiac magnetic resonance imaging (CMR) in children with acute left ventricular (LV) dysfunction, specifically assessing end-diastolic volume (EDV), end-systolic volume (ESV), and left ventricular ejection fraction (LVEF) and to identify any associated systematic measurement errors.

Methods

An observational cohort study was conducted in New Delhi, India, over a period of 18 months from June 2021 to November 2022. A total of 16 children aged one month to 18 years with acute myocarditis or new-onset LV dysfunction were enrolled. Clinical details, laboratory parameters, echocardiography, and CMR findings were recorded. Correlations were assessed using Pearson’s coefficient, and agreement was analyzed using Bland-Altman plots.

Results

A very strong positive correlation was found between 3D-ECHO and CMR for LVEF (r = 0.887; p < 0.0001). EDV and ESV also showed strong correlations between 3D-ECHO and CMR (r = 0.906 and r = 0.903, respectively). However, the Bland-Altman analysis revealed that 3D-ECHO consistently underestimated EDV (mean difference = -18.86; p = 0.034) and ESV (mean difference = -21.77; p = 0.014) compared to CMR, while LVEF showed good agreement (p = 0.619).

Conclusion

3D-ECHO demonstrates strong correlation with CMR in assessing LVEF in children with acute LV dysfunction and may serve as a reliable bedside tool for systolic function evaluation. However, its underestimation of EDV and ESV highlights the continued role of CMR as the gold standard for precise volumetric assessment.

## Introduction

The prevalence of left ventricular (LV) dysfunction in the general population has been estimated at around 1% [1]. The most common causes of heart failure in adults are attributed to coronary artery disease and hypertension, whereas those in the pediatric population involve congenital heart diseases (CHD) and cardiomyopathies [2]. The most common cause of acute LV dysfunction in the pediatric population has been determined to be myocarditis, which accounted for approximately 0.05% of all pediatric admissions to tertiary care hospitals [3]. Despite its rarity, myocarditis is a significant etiology of acute and chronic heart failure, often leading to dilated cardiomyopathy (DCM) and the need for heart transplantation [4]. Although DCM may result from other etiologies, such as neuromuscular disorders, inborn errors of metabolism, or other familial syndromes, a recent study demonstrated that 27%-46% of cases of DCM were secondary to viral myocarditis [5].

The assessment of ventricular volume among the pediatric population has always been a significant challenge because of the complications, especially in the right ventricle [6]. Two-dimensional echocardiography (2D-ECHO) has been a feasible and low-cost option [7]. However, it rarely provides assumptions of ventricular volumes. For the volumetric analysis, cardiac magnetic resonance imaging (CMR) has been the gold standard approach, especially for the evaluation of the right ventricle [8,9]. However, its use is limited in the pediatric population owing to the time and cooperation needed from them, especially in hyperactive or small children, where general anesthesia may be needed and imposes potential risks.

Three-dimensional echocardiography (3D-ECHO) has been an accessible option for evaluating cardiac morphology in routine cardiology practice [10,11]. Not only does it combine the cost-effectiveness and accessibility of 2D echocardiography, but also it inculcates the accuracy as provided by CMR, which emphasizes that it can be a useful modality for evaluating ventricular volumes in the pediatric population to evaluate the cases of LV dysfunction [12].

Existing literature indicates a strong concordance between 3D-ECHO and CMR, particularly for the assessment of ventricular systolic function [13,14]. Nonetheless, findings related to volumetric measurements in pediatric populations remain inconclusive. Furthermore, there is a lack of published data directly comparing biventricular volumes obtained through 3D-ECHO and validated against CMR within the same cohort of patients [6].

Thus, this study was done to evaluate the correlation in findings of 3D-ECHO and CMR in children with acute LV dysfunction. The objectives were to find the correlation and agreement between 3D-ECHO and CMR in children with acute left ventricular dysfunction, specifically assessing end-diastolic volume (EDV), end-systolic volume (ESV), and left ventricular ejection fraction (LVEF), and to identify any associated systematic measurement errors.

## Materials and methods

An observational cohort study was carried out in the department of pediatric cardiology at Fortis Escorts Heart Institute, New Delhi, a tertiary care health center in India, over a period of 18 months from June 2021 to November 2022. The study population comprised children aged one month to 18 years, fulfilling the inclusion and exclusion criteria. The Institutional Ethics Committee of Fortis Escorts Heart Institute issued approval IEC/2021/OAS/03.

Inclusion criteria

Patients aged one month to 18 years presenting with a diagnosed case of acute myocarditis or new-onset left ventricular dysfunction must have at least one of the following: clinical symptoms (fever, viral prodrome, chest pain, palpitations, or dyspnea) occurring within six weeks of presentation, elevation of at least one myocardial damage biomarker (troponin T/I and creatine phosphokinase {CPK}/CPK-myocardial band {MB}), or echocardiographic evidence of structural or functional myocardial abnormalities.

Exclusion criteria

The exclusion criteria were as follows: caregivers who do not provide consent, patients with suboptimal transthoracic echocardiographic acoustic windows, and children with any congenital heart disease, previous cardiac surgery, or known coronary artery disease.

Sample size

Kamińska et al. reported strong correlations between 3D echocardiography and cardiac magnetic resonance imaging (CMR) measurements for LVEDV and LVESV, with correlation coefficients of 0.892 and 0.896, respectively [6]. Based on these values, the minimum required sample size was calculated to be 10 patients at a 95% power and a 5% level of significance. To enhance the reliability of the results and minimize potential error, a total of 16 patients were included in the study.

Procedure

The study was explained to the caregivers of study subjects in the local language with the help of the information sheet. Assent was taken from children aged >7 years in addition to written consent from the parents or legal guardians for children less than seven years. All the data was recorded onto the study proforma.

Patient details noted were age, gender, height, weight, body mass index (BMI), and body surface area (BSA). Details of previously diagnosed conditions, prior hospital admissions, and family history were noted. Clinical symptoms of the patients were also noted. A short medical history was obtained from the parents/guardian to assess the patient’s state of health and any current concerns. Further history was taken to establish any previously diagnosed illness or any significant family history. A careful history of SARS-CoV-2 in the patient or any of the patient’s contacts was also extracted. A complete physical examination was then carried out. The laboratory biochemical parameters were recorded in the study proforma. The results from 2D-ECHO, 3D-ECHO, and CMR were obtained. Echocardiography was performed using the Philips Epiq 7® (Philips, Amsterdam, Netherlands) machine with different probe sizes. CMR was done using Philips Achieva 1.5T® using different protocols. The same individual (primary investigator) performed 3D-ECHO and CMR.

The primary outcome was the correlation between LVEF measured by 3D-ECHO and CMR. Secondary outcomes included the comparison and correlation of EDV and ESV measured by 3D-ECHO, 2D-ECHO, and CMR.

Statistical analysis

Categorical variables were summarized as frequencies and percentages, whereas continuous variables were expressed as mean ± standard deviation (SD) for normally distributed data or as median with interquartile range (IQR) for non-normally distributed data, as determined by the Shapiro-Wilk test. Statistical analysis involved Pearson’s correlation coefficient to evaluate relationships among LVEF, EDV, and ESV measured by 2D, 3D, and magnetic resonance imaging (MRI) methods, while Bland-Altman plots were used to assess agreement between 3D echocardiography and MRI. Data were entered into Microsoft Excel (Microsoft Corp., Redmond, WA) and analyzed using SPSS software version 25.0 (IBM Corp., Armonk, NY). A p-value of less than 0.05 was considered statistically significant.

## Results

The median age of the study subjects was 72 (22.5-126) months, with a median weight of 17.8 (10.325-28) kg, mean height of 111.56 ± 33.23 cm, mean BMI of 14.32 ± 5.48 kg/m², and mean BSA of 0.78 ± 0.38 m². There was an equal gender distribution with eight (50%) women and eight (50%) men. For weight for age, three (18.75%) were at the third centile, three (18.75%) at the fifth, four (25%) at the 25th, three (18.75%) at the 50th, two (12.5%) at the 90th, and one (6.25%) at the 97th centile. For height for age, five (31.25%) each were at the 25th and 50th centiles, two (12.5%) at the 75th, three (18.75%) at the 90th, and one (6.25%) at the 97th centile. Previously diagnosed conditions included dilated cardiomyopathy, pulmonary tuberculosis (TB), and systemic lupus erythematosus (SLE) in one (6.25%) case each, while four (25%) had a history of previous admission, and one (6.25%) had a positive family history. Clinical symptoms included cough in 12 (75%), dyspnea in 11 (68.75%), fever and easy fatiguability in nine (56.25%) each, gastrointestinal (GI) symptoms in seven (43.75%), swelling over the body in six (37.5%), sweating in four (25%), chest pain in three (18.75%), and rash in one (6.25%), while palpitation, periungual swelling, and peeling of skin were absent (Table 1).

**Table 1:** Baseline demographic and clinical characteristics of the study cohort Values are in n (%)/mean ± SD (range)/median (25th-75th percentile) BSA, body surface area; DCM, dilated cardiomyopathy; TB, tuberculosis; SLE, systemic lupus erythematosus; GI, gastrointestinal; SD, standard deviation

Demographic characteristics	Values
Age (months)	72 (22.5-126)
Weight (kg)	17.8 (10.325-28)
Height (cm)	111.56 ± 33.23 (62-156)
Body mass index (kg/m²)	14.32 ± 5.48 (0.78-26.3)
BSA (m²)	0.78 ± 0.38 (0.28-1.6)
Gender	
Female	8 (50.00%)
Male	8 (50.00%)
Previously diagnosed conditions	
DCM	1 (6.25%)
Pulmonary TB	1 (6.25%)
SLE	1 (6.25%)
Previous admissions	4 (25.00%)
Family history	1 (6.25%)
Clinical symptoms	
Fever	9 (56.25%)
GI symptoms	7 (43.75%)
Cough	12 (75.00%)
Dyspnea	11 (68.75%)
Swelling over the body	6 (37.50%)
Palpitation	0 (0.00%)
Sweating	4 (25.00%)
Easy fatiguability	9 (56.25%)
Chest pain	3 (18.75%)
Rash	1 (6.25%)

LVEF, EDV, and ESV were determined on the three modalities, the values of which are shown in Table 2.

**Table 2:** Descriptive statistics of LVEF, EDV, and ESV of study subjects Values are in mean ± SD (range) LVEF, left ventricular ejection fraction; EDV, end-diastolic volume; ESV, end-systolic volume; 2D-ECHO, two-dimensional echocardiography; 3D-ECHO, three-dimensional echocardiography; CMR, cardiac magnetic resonance imaging; SD, standard deviation

Parameters	2D-ECHO	3D-ECHO	CMR
LVEF (%)	20.77 ± 6.63 (10-34)	19.62 ± 6.93 (9.9-33)	19.03 ± 9.48 (7.6-37)
EDV (mL)	108.03 ± 51.37 (40-220)	124.15 ± 57.76 (35-240)	143.01 ± 73.38 (30-300)
ESV (mL)	84.07 ± 44.24 (18-180)	97.31 ± 47.57 (26-208)	119.08 ± 66.58 (19-268)

Correlation analysis

**Figure 1:**
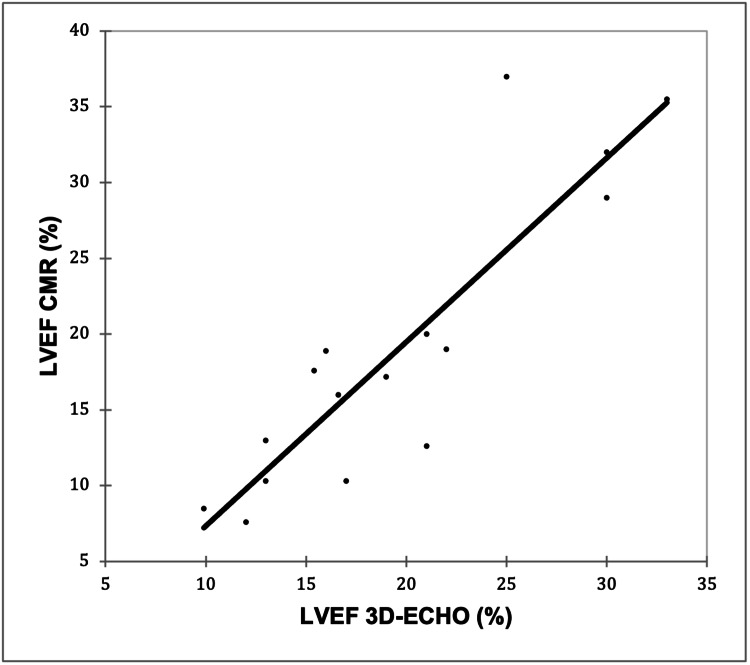
Correlation of LVEF 3D-ECHO with LVEF CMR Pearson’s correlation coefficient (r) = 0.887; p < 0.0001 (p < 0.05 was considered statistically significant) LVEF, left ventricular ejection fraction; 3D-ECHO, three-dimensional echocardiography; CMR, cardiac magnetic resonance imaging

A very strong positive correlation was observed between LVEF 3D-ECHO and LVEF CMR (r = 0.887; p < 0.0001), as shown in Figure 1.

**Figure 2:**
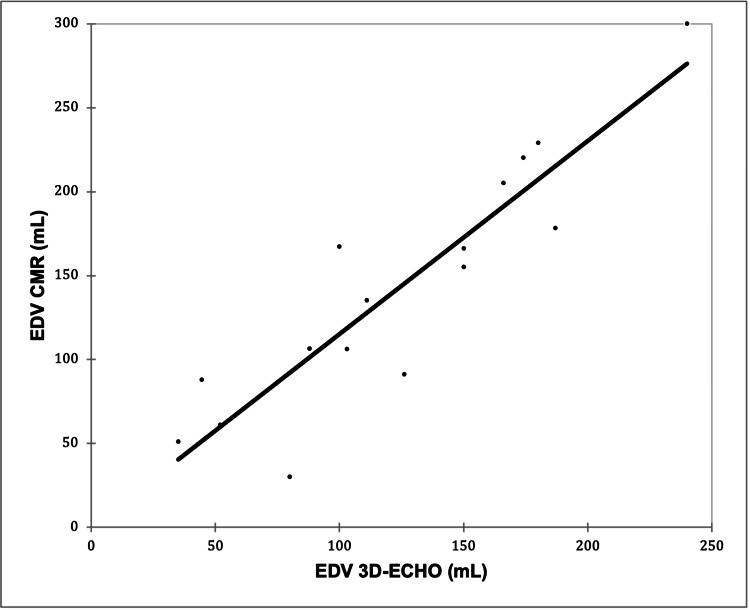
Correlation of EDV 3D-ECHO with EDV CMR Pearson’s correlation coefficient (r) = 0.906; p < 0.0001 (p < 0.05 was considered statistically significant) EDV, end-diastolic volume; 3D-ECHO, three-dimensional echocardiography; CMR, cardiac magnetic resonance imaging

A very strong positive correlation was observed between EDV 3D-ECHO and EDV CMR (r = 0.906; p < 0.0001), as shown in Figure 2.

**Figure 3:**
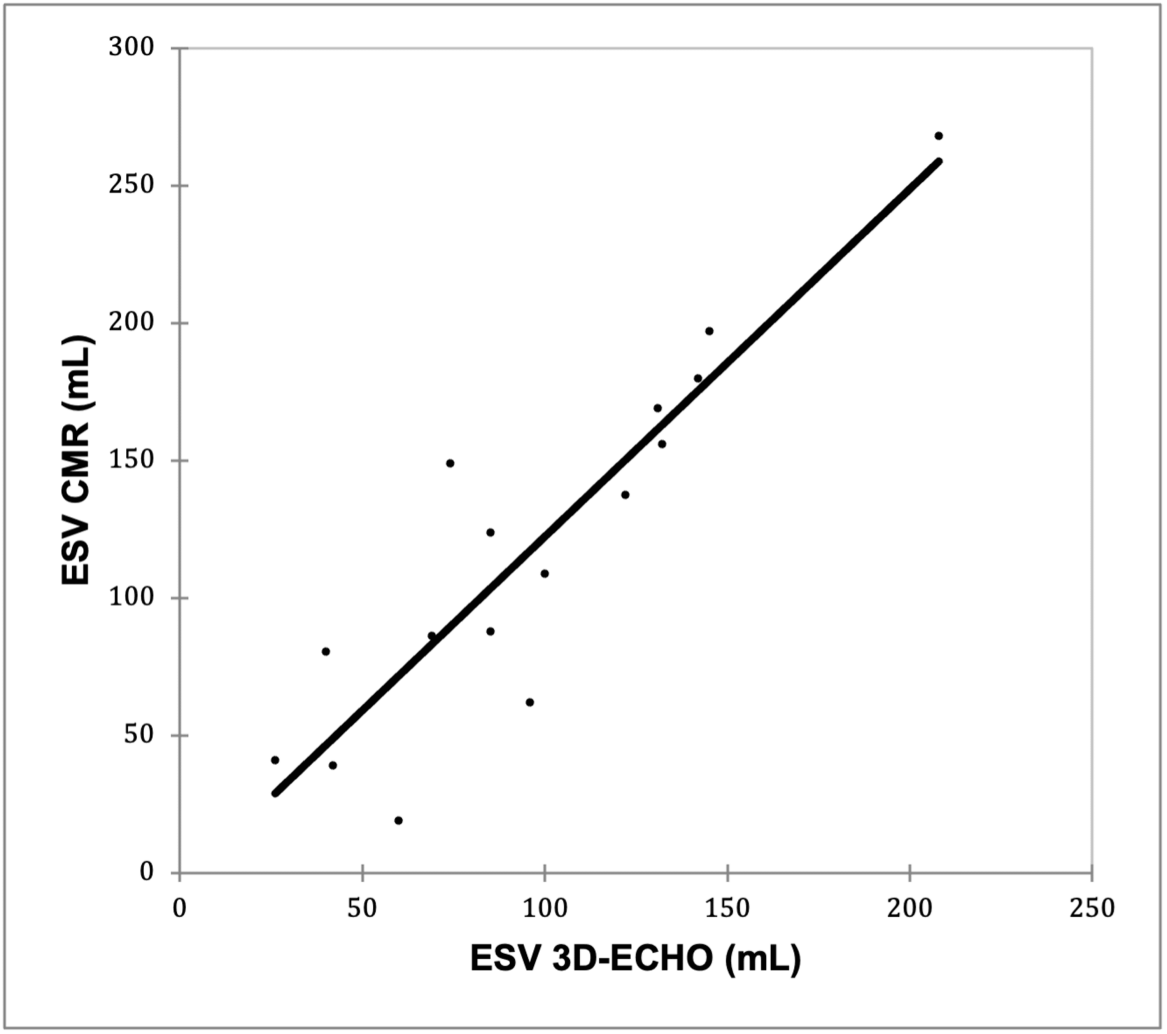
Correlation of ESV 3D-ECHO with ESV CMR Pearson’s correlation coefficient (r) = 0.903; p < 0.0001 (p < 0.05 was considered statistically significant) ESV, end-systolic volume; 3D-ECHO, three-dimensional echocardiography; CMR, cardiac magnetic resonance imaging

A very strong positive correlation was observed between ESV 3D-ECHO and ESV CMR (r = 0.903; p < 0.0001), as shown in Figure 3.

Agreement analysis

**Figure 4:**
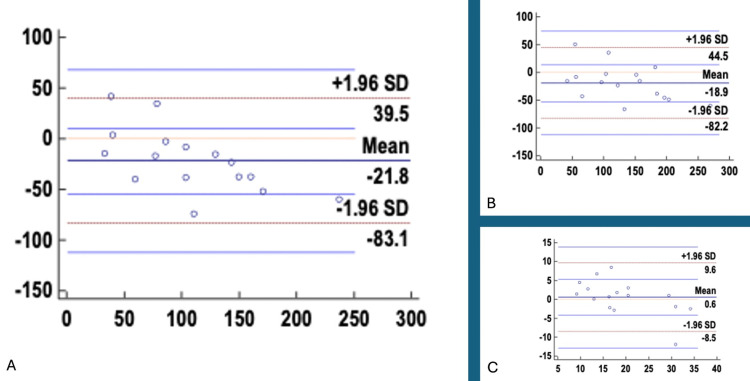
Bland-Altman Plots comparing 3D-ECHO and CMR parameters: (A) ESV, (B) EDV, and (C) LVEF LVEF, left ventricular ejection fraction; EDV, end-diastolic volume; ESV, end-systolic volume; 3D-ECHO, three-dimensional echocardiography; CMR, cardiac magnetic resonance imaging; SD, standard deviation

The Bland-Altman analysis between 3D echocardiography and CMR demonstrated good overall agreement for LVEF, with a mean difference of 0.59 that was not statistically significant (p = 0.619), indicating no systematic bias between the two modalities. However, for left ventricular EDV and ESV, 3D echocardiography showed statistically significant underestimation compared to CMR, with mean differences of -18.86 (p = 0.034) and -21.77 (p = 0.014), respectively. The wide limits of agreement observed for EDV and ESV suggest variability between methods, particularly for volume measurements (Table 3 and Figure 4).

**Table 3:** Bland-Altman analysis comparing 3D-ECHO and CMR parameters P < 0.05 was considered statistically significant CI, confidence interval; LVEF, left ventricular ejection fraction; EDV, end-diastolic volume; ESV, end-systolic volume; 3D-ECHO, three-dimensional echocardiography; CMR, cardiac magnetic resonance imaging

Parameter	Sample size (n)	Mean difference	95% CI (mean)	P-value	Standard deviation	Lower limit of agreement	95% CI (lower)	Upper limit of agreement	95% CI (upper)
LVEF	16	0.59	-1.88 to 3.05	0.619	4.62	-8.47	-12.78 to -4.17	9.65	5.35 to 13.95
EDV	16	-18.86	-36.08 to -1.64	0.034	32.32	-82.20	-112.27 to -52.13	44.48	14.41 to 74.55
ESV	16	-21.77	-38.43 to -5.11	0.014	31.27	-83.06	-112.15 to -53.96	39.52	10.43 to 68.62

## Discussion

The study remains significant in assessing 3D-ECHO and CMR among 16 children with an equivalent male/female distribution of 1:1. Primarily, three parameters, namely, LVEF, EDV, and ESV, were assessed, and it was found that there was a significant positive correlation between 3D-ECHO and CMR, with p < 0.0001. However, when a Bland-Altman plot was made, it was found that only LVEF values showed good agreement between CMR and 3D-ECHO, while ESV and EDV showed significant variation with p < 0.05. There was a wide limit of agreement ranging from -82.2 to 44.48 for EDV and -83.05 to 39.56 for ESV. This shows that 3D-ECHO may not be aptly applied in terms of volumetric assessments (EDV and ESV), while it may show similarity in functional assessment (LVEF) with CMR in children with acute LV dysfunction.

Our findings align with Kamińska et al., who assessed 43 children using 3D-ECHO and CMR [6]. They reported strong correlations for left ventricular volumes: LVEDV (r = 0.892; p < 0.00001) and LVESV (r = 0.896; p < 0.00001), with the Bland-Altman analysis showing narrow limits of agreement. Mean LVEF values were nearly identical between modalities, 58% by 3D-ECHO versus 59% by CMR, underscoring the reliability of ejection fraction (EF) assessment. In contrast, right ventricular (RV) volumes were systematically underestimated by 3D-ECHO: RVEDV by 38% (104.40 ± 49.52 mL versus 153.08 ± 64.28 mL; p < 0.000001) and RVESV by 45% (48.39 ± 29.07 mL versus 73.29 ± 42.90 mL; p < 0.00001), with wide Bland-Altman limits. To address this, correction coefficients of 1.38 (RVEDV) and 1.45 (RVESV) significantly improved agreement. These findings highlight 3D-ECHO’s reliability for LVEF but limitations in volumetric indices. Taken together, these results emphasize that while 3D-ECHO can be reliably used to assess systolic function parameters such as LVEF in children, caution is warranted in interpreting absolute volumetric indices such as EDV and ESV, as significant intermodality variation may occur.

Our findings can also be compared with those of Liu et al, who validated fully automated 3D-ECHO against CMR in 82 children [15]. Their study reported strong correlations for right ventricular volumes and ejection fraction: RVEDV, r = 0.93; RVESV, r = 0.90; and RVEF, r = 0.82 (all p < 0.001). Although 3D-ECHO underestimated RV volumes (RVEDV, 71.4 mL versus 85.1 mL; RVESV, 34.4 mL versus 45.3 mL) and overestimated RVEF (48.9% ± 13.5% versus 42.2% ± 14.2%), the Bland-Altman analysis showed acceptable limits of agreement (RVEDV, -63.4 to 41.5 mL; RVESV, -53.7 to 28.0 mL; and RVEF, -9.7% to 23.0%). These findings parallel our results, where a significant positive correlation between 3D-ECHO and CMR was noted, yet wide limits of agreement persisted for EDV and ESV, highlighting that 3D-ECHO provides reliable LVEF estimates but limited volumetric precision in pediatric ventricular dysfunction.

Friedberg et al. evaluated neonates and infants with congenital heart disease and similarly reported a high correlation between 3D-ECHO and CMR for LV volumes and mass (intraclass correlation coefficient {ICC} for EDV 0.96 and for ESV 0.90) but with systematic differences [16]. Their study demonstrated that EDV measured by 3D-ECHO was comparable to MRI (mean difference, -0.49 mL; p = 0.6), whereas ESV values were consistently higher by 2.7 mL (p = 0.001). Ejection fraction by 3D-ECHO was lower than CMR by approximately 9.3% (p = 0.0004). Thus, while Friedberg et al. concluded that 3D-ECHO provides reliable estimates of LV volumes and mass in neonates and infants with complex CHD, the underestimation of EF compared to MRI contrasts with our findings, where LVEF showed the best agreement between modalities [16]. Conversely, in our study, EDV and ESV showed poor agreement, whereas Friedberg et al. demonstrated relatively close alignment of EDV with CMR and only mild overestimation of ESV [16]. These differences may be due to differences in patient age, ventricular geometry, and disease pathology, as our study included older children with acute LV dysfunction, while their study included neonates and infants with congenital structural abnormalities.

In contrast to our observations, Habeeb et al. evaluated 20 children with dilated cardiomyopathy and reported no significant differences between 3D-ECHO and CMR in the assessment of volumetric parameters [17]. Their results showed close agreement, with mean EDV/BSA of 92.69 ± 27.44 mL/m² by 3D-ECHO and 93.41 ± 27.33 mL/m² by CMR and mean ESV/BSA of 58.58 ± 23.89 mL/m² by 3D-ECHO and 58.65 ± 24.11 mL/m² by CMR. Similarly, LVEF values were nearly identical, being 40.25% ± 7.65% by 3D-ECHO and 39.56% ± 9.80% by CMR (p = 0.996). These findings indicate strong concordance across modalities. While both studies suggest that 3D-ECHO can reliably approximate CMR for ejection fraction, our results diverge with respect to EDV and ESV, which demonstrated significant variation and poor agreement on the Bland-Altman analysis, limiting the use of 3D-ECHO in the context of acute pediatric LV dysfunction.

It must be reasoned out here that in children, a smaller body size enables better visualization of the ventricles, leading to reduced intra- and inter-observer variability and improved measurement reliability [6]. The application of matrix array transducers and semiautomatic tracking software enhances the precision of endocardial border detection, reducing human bias and minimizing the underestimation of ventricular cavity size. Additional refinements, such as high-contrast monitor settings and the manual extraction of the left ventricular outflow tract, further enhance the accuracy of volumetric assessment. These methodological improvements allow 3D-ECHO to produce left ventricular volumes and ejection fractions that show excellent correlation and agreement with CMR. Although 3D-ECHO underestimated EDV and ESV, the error remains proportional across measurements, preserving correlation even when absolute values differ [6]. Consistent acquisition conditions and validated correction coefficients may further strengthen intermodality reliability between 3D-ECHO and CMR in pediatric ventricular volume assessment [6,15].

Although our findings demonstrate a high level of agreement between 3D-ECHO and CMR for ejection fraction, this relationship must be interpreted with caution. An agreement in EF can occur even when systematic errors exist in the underlying volume measurements due to acoustic window limitations, geometric assumptions, and lower endocardial border resolution. If these systematic biases occur in a proportionally similar manner in both EDV and ESV, the calculated EF may appear concordant despite persistent absolute volume discrepancies. In our dataset, the Bland-Altman analysis indicated variability in EDV and ESV with potential fixed and proportional biases, suggesting that the high EF agreement should not be interpreted as complete interchangeability between methods. Recognizing these systematic measurement differences is essential for understanding the clinical implications of modality selection, particularly in pediatric patients with acute left ventricular dysfunction where small volume errors may influence clinical decision-making.

Limitations of the study

This study’s limitations include a small sample size of 16 participants and its single-center design. Since patients were not followed up, the assessment of long-term outcomes could not be done. Excluding patients with poor acoustic windows may have introduced selection bias, and the inclusion of only acute LV dysfunction cases limits applicability to chronic or other cardiac conditions.

## Conclusions

In conclusion, this study showed that 3D-ECHO correlates well with CMR for assessing left ventricular function in children, particularly LVEF, confirming its reliability as a noninvasive clinical tool. However, 3D-ECHO consistently underestimates ventricular volumes (EDV and ESV) compared to CMR, indicating limitations in volumetric assessment. While useful for the bedside evaluation of systolic function, CMR remains the reference standard for precise volumetric measurements.
